# A Giant Internal Carotid Bifurcation Aneurysm as a Rare and Dangerous Differential Diagnosis of a Craniopharyngioma

**DOI:** 10.7759/cureus.21588

**Published:** 2022-01-25

**Authors:** Caio M Perret, Raphael Bertani, Stefan W. Koester, Paulo Eduardo Santa Maria, Daniela Von Zuben, Sávio Batista, Hugo C Schiavini, José Alberto Landeiro

**Affiliations:** 1 Neurological Surgery, Hospital Municipal Miguel Couto, Rio de Janeiro, BRA; 2 Neuroscience, Federal University of Rio de Janeiro, Rio de Janeiro, BRA; 3 Neurosurgery, Hospital Municipal Miguel Couto, Rio de Janeiro, BRA; 4 Neurosurgery, Vanderbilt University School of Medicine, Nashville, USA; 5 Faculty of Medicine, Federal University of Rio de Janeiro, Rio de Janeiro, BRA; 6 Neurosurgery, Hospital Universitario Antonio Pedro, Rio de Janeiro, BRA

**Keywords:** giant carotid aneurysm, imaging, carotid bifurcation aneurysm, craniopharyngioma, giant aneurysm

## Abstract

Craniopharyngiomas are supra/parasellar lesions that often present with general, unspecific symptoms. Similarly, internal carotid artery (ICA) bifurcation giant aneurysms may also produce calcified, heterogeneous, parasellar expansive lesions, posing a relevant differential diagnosis due to their inherently different surgical strategies and risks. We report the case of a 54-year-old female presenting with progressive disorientation and apathetic behavior. CT and MRI reports described a suprasellar heterogenous mass with calcifications associated with an adjacent, laterally located fluid collection suggestive of a craniopharyngioma. During the surgical procedure, perfuse and unexplained arterial bleeding ensued, prompting the surgical team to review a previous contrast-enhanced CT scan. Careful inspection revealed an image suggestive of vascular pathology, with an area of continuous hyperdensity along the right ICA bifurcation. The Sylvian fissure was dissected, and an aneurysmal neck was encountered and successfully clipped. Giant intracranial aneurysms are rare but essential differential diagnoses to be considered during the workup and surgical approach toward parasellar mass lesions. This case illustrates the importance of performing a CT angiogram (CTA) for skull base lesions, even when the size is more suggestive of tumor pathology.

## Introduction

The differential diagnosis of lesions of the sellar, parasellar, and suprasellar regions of the central nervous system (CNS) includes various lesions with varying etiologies. Not all of them are neoplastic (and more recurrent), such as the more common Rathke cleft cysts, pituitary adenomas, and craniopharyngiomas. There are granulomatous processes, autoimmune diseases, and immunocompromised state-related infections among rare cases. These cases potentially impose significant challenges in establishing the proper diagnosis and clinical conduction because of the low frequency in this anatomic region. They may present similar signs, symptoms, and radiological findings [1]. Therefore, as a therapeutic approach, the diagnostic conclusion of these lesions is only adequate and thorough after histopathological studies, which require surgical intervention and biopsy of the lesion [2].

Craniopharyngiomas are calcified, cystic, or mixed solid and cystic tumors. Embryologically, they originate from Rathke's pouch and extend into the suprasellar midline region, often directed into the third ventricle, along the pituitary stalk and the optic chiasm [3]. Main neuroimaging findings include calcifications in the parasellar region, cystic degeneration, and sometimes distortions of the sellar anatomy. Due to their location, these lesions may produce hormonal imbalances and vision loss, apart from more general symptoms such as chronic headache, depression, and disorientation [4,5].

However, some lesions might not be amenable to a straightforward diagnosis due to their unusual presentation on simple imaging, and their true nature is only revealed either with a more detailed workup with vascular studies or only intraoperatively. In addition, carotid bifurcation giant aneurysms may also produce calcified, heterogeneous, parasellar expansion-masses mimicking the appearance of a craniopharyngioma, posing a relevant differential due to their inherently different surgical strategies and risks [6]. In the entire medical literature, two papers have reported giant aneurysms mimicking craniopharyngiomas both clinically and radiologically [7,8].

We report the case of a patient presenting with a heterogeneous, calcified, parasellar/suprasellar mass lesion measuring 5.1 cm, with an apparent 6.0-cm cystic-degeneration; it was reported as a probable craniopharyngioma on a radiologist's notes, and which was revealed intraoperatively to be a giant, calcified aneurysm upon its inadvertent incision, subsequent bleeding, and successful treatment with direct clipping.

## Case presentation

**Figure 1:**
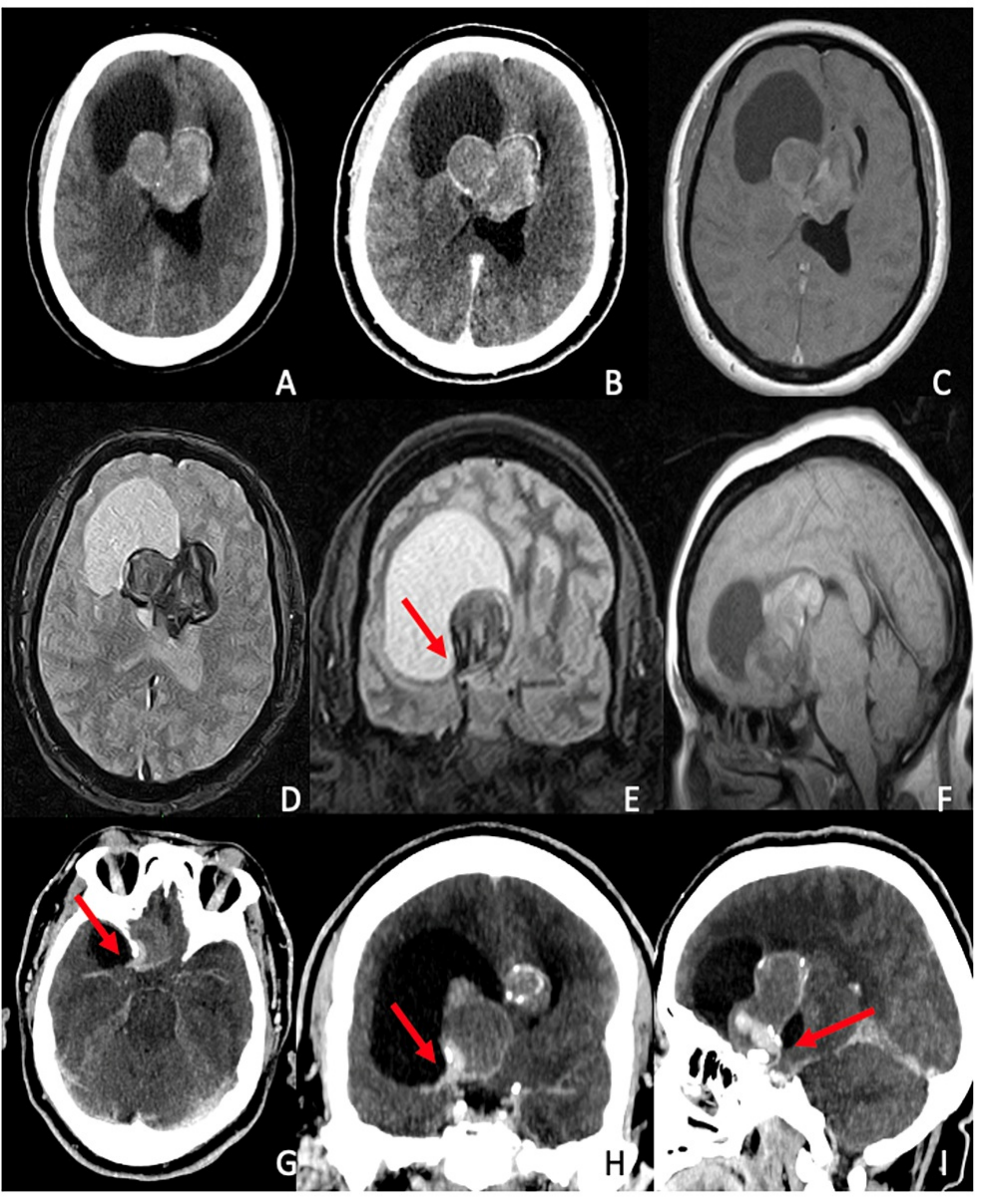
Preoperative neuroimaging assessments Axial section contrast-enhanced CT scan showed a midline mass with peripheral calcifications (A) and heterogeneous contrast enhancement (B). The right frontal cystic lesion appears more hyperintense than the CSF in contrast-enhanced MRI sequences. SWI reconstructions evidenced flow voids (D, E) and heterogenous contrast enhancement is seen on sagittal T1 (F). Prior radiology report suggested craniopharyngioma. A contrast-enhanced CT scan adjusted for arterial vessels evidenced a hyperdense pattern within the tumoral wall, suggesting luminal patency (red arrows) from the right ICA bifurcation CT: computed tomography; CSF: cerebrospinal fluid; MRI: magnetic resonance imaging; SWI: susceptibility-weighted imaging; ICA: internal carotid artery

A 54-year-old female patient presented to the emergency room with progressive disorientation, apathetic behavior, and bilateral lower limb weakness. She underwent a CT scan to rule out bleeding, which was followed by a contrast-enhanced CT scan when a suprasellar mass was identified. MRI showed a suprasellar heterogenous mass with calcification areas associated with an adjacent, lateralized fluid collection. Intraoperatively, further contrasted CT reconstruction evidenced a hyperdense image within the tumor wall, continuous with the top of the right carotid artery. The radiological report suggested a craniopharyngioma (Figure 1). The patient had a clinical history of hypothyroidism, with no other hormonal or metabolic alterations in the preoperative screening.

An extended pterional incision and a fronto-orbitozygomatic approach for the resection of the tumor were planned. A middle frontal gyrus corticectomy was performed to gain access to the cystic portion of the lesion. The cyst was drained, and a firm lesion was noticed in the cavity. The lesion was dissected and debulked for volume reduction and easier maneuverability.

**Figure 2:**
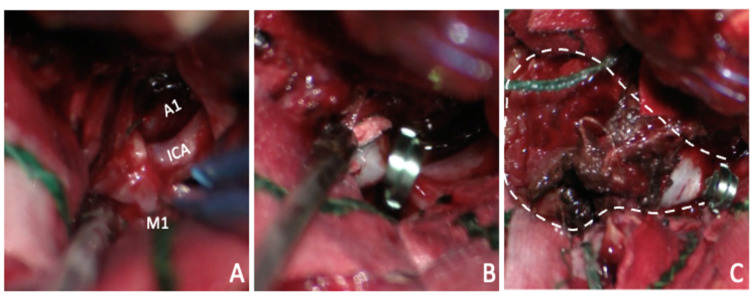
Intraoperative microscopic view of the lesion dissection Microscopic view of the aneurysm neck dissection (A) and clipping (B). Wide exposure, neck, and opened dome with thrombus and hemostats inside (traced line) and final clip position (C)

During debulking, high-volume arterial bleeding ensued. It was not compatible with the injury of a standard, tumor-related artery. Hemostasis was achieved with fibrillar hemostats and irrigation under pressure. The high-flow bleeding and location of the lesion raised suspicion for an aneurysmal lesion of the internal carotid artery (ICA). The Sylvian fissure was dissected immediately, and an aneurysmal neck was encountered at the ICA bifurcation (Figure 2) and clipped with a 13-mm straight clip, ceasing the arterial flow. Thrombectomy was performed. The event lasted for approximately 15 minutes and caused momentary hemodynamic instability that was corrected shortly after clipping.

## Discussion

Giant intracranial aneurysms are arterial malformations greater than 2.5 cm that represent only 2-5% of all intracranial aneurysms [6,9]. They usually present between the fifth and the seventh decade of life, with a slight predominance among the female sex. Giant aneurysms may impose significant challenges in surgical management. Due to their increased size, symptoms associated with mass effect may be seen with unruptured aneurysms, related to their location. Hence, they should be considered in the differential diagnosis of expansion lesions of the CNS [1,6].

As expansion lesions, giant aneurysms might present cystic, degenerated, heterogeneous, lobulated, and calcified lesions when unruptured [7,8]. Due to the parasellar topography, giant carotid aneurysms might show sufficient mass effects to cause pituitary and hypothalamic dysfunction, as seen in this case [4]. There are two reports in the medical literature in which giant aneurysms were previously misdiagnosed as craniopharyngiomas [7,8].

Due to limited resources and the patient being admitted with contrasted imaging suggestive of a tumor, a CT angiogram (CTA) was not performed since it was not deemed essential by the hospital's administration. However, we believe that this type of advanced vascular imaging would have helped diagnose the aneurysm before the surgery.

## Conclusions

We reported a case of a giant aneurysm presenting as a parasellar/suprasellar lesion that resembled a craniopharyngioma. Advanced vascular imaging would likely have helped with the proper diagnosis of the lesion. The radiological findings and the unspecific symptom onset point to later diagnoses in these reported cases, with their true natures being unveiled only during intraoperative dissection or during preoperative vascular assessments when available or considered. Although our case was resolved without nefarious consequences, knowing what pathology was being operated on would have allowed for more adequate surgical planning and avoided significant intraoperative hemorrhage and potential harm. We recommend that surgeons increase their efforts in requesting advanced vascular imaging when faced with lesions of the sellar/parasellar regions, as there may be more to them than meets the eye.
